# Case presentation of 8-year follow up of recurrent malignant duodenal Insulinoma and lymph node metastases and literature review of malignant Insulinoma management

**DOI:** 10.1186/s12902-022-01219-9

**Published:** 2022-12-09

**Authors:** Michelle P. Walker, Vikram Shenoy, David C. Metz, Charles A. Stanley, Douglas Fraker, Vinay Chandrasekhara, Anastassia Amaro

**Affiliations:** 1 Department of Endocrinology, University of Pennsylvania, 3400 Civic Center Boulevard, West Pavilion, 4th Floor, Philadelphia, PA 19104 USA; 2 Department of Endocrinology, Christiana Care, 4735 Ogletown Stanton Road, Suite MAP2, Newark, DE 19713 USA; 3 Department of Gastroenterology, University of Pennsylvania, 3400 Civic Center Boulevard, South Pavilion, 4th Floor, Philadelphia, PA 19104 USA; 4 Department of Endocrinology and Metabolism, Children’s Hospital of Philadelphia, 3516 Civic Center Boulevard #802, Philadelphia, PA 19104 USA; 5 Department of Endocrine and Oncologic Surgery, University of Pennsylvania, 3400 Civic Center Boulevard, West Pavilion, 3rd Floor, Philadelphia, PA 19104 USA; 6 Department of Gastroenterology and Hepatology, Mayo Clinic, 200 First Street. SW, Rochester, MN 55905 USA

**Keywords:** Extrapancreatic insulinoma, Ectopic insulinoma, Insulinoma recurrence, Case presentation

## Abstract

**Background:**

Insulinoma is an uncommon insulin-secreting neuroendocrine tumor that presents with severe recurrent hypoglycemia. Although cases of extrapancreatic insulinomas have been reported, the majority of insulinomas occur in the pancreas. The number of reported cases of ectopic insulinomas with follow-up assessments is limited and they do not report disease recurrence. The current report presents the first documented case of recurrent extrapancreatic insulinoma with 8 years of follow-up, provides relevant literature review, and proposes surveillance and treatment strategies.

**Case presentation:**

We describe an insulinoma localized in the duodenal wall of a 36-year-old female who presented in 2013 with weight gain and Whipple’s triad and was successfully managed with duodenotomy and enucleation. She presented again in 2017 with recurrent Whipple’s triad and was found to have metastatic disease localized exclusively to peripancreatic lymph nodes. Primary pancreatic insulinoma was not evident and her hypoglycemia resolved following lymph node dissection. Eight years after initial presentation continuous glucose monitoring (CGM) showed a trend for euglycemia, and PET-CT Gallium 68 DOTATATE scan evaluation indicated absence of recurrent disease.

**Conclusion:**

Insulinomas are rare clinical entities and extrapancreatic insulinomas are particularly uncommon. Follow-up evaluation and treatment strategies for ectopic insulinoma recurrence presents a significant clinical challenge as the condition has hitherto remained undescribed in the literature. Available evidence in the literature indicates that lymph node metastases of intrapancreatic insulinomas likely do not change prognosis. Given the absence of long-term data informing the management and monitoring of patients with extrapancreatic insulinoma, we suggest patient education for hypoglycemic symptoms, monitoring for hypoglycemia with CGM, annual imaging, and a discussion with patients regarding treatment with octreotide or alternative somatostatin receptor analog therapies.

## Background

Insulinoma is a rare neuroendocrine tumor (NET) with an incidence of 0.4 cases per 100,000 per year, and is typically seen in the 5th decade of life with a slight predominance of the female sex [1]. The vast majority of these cases (> 85%) are benign and almost all occur within the pancreatic parenchyma; however, ectopic insulinomas have previously been described in the duodenum [2], duodenohepatic ligament [3], kidney [4], appendix [5], spleen [6], perisplenic tissue [7], and adjacent to the ligament of Treitz [8]. Each of these extrapancreatic insulinoma cases was successfully managed with surgery, but the longest recurrence-free follow-up time described was only 3 months with no further reported follow-up assessment.

In the current report, we describe a 36-year-old female who presented with a primary insulinoma in the wall of the second portion of the duodenum that was surgically managed with resolution of her hypoglycemia. She then presented 4 years later with recurrent hypoglycemia and localized peripancreatic lymph node disease. We provide 8-year follow-up results and discuss strategies for follow-up evaluation, surveillance, and management of recurrent, metastatic extrapancreatic insulinoma.

## Case presentation

In June 2013, a 36-year-old female presented with Whipple’s triad (documented, symptomatic hypoglycemia that responded to glucose) and weight gain of 40 pounds. Her serum glucose level was 49 mg/dL (normal range: 70–99 mg/dL) with inappropriately elevated insulin level of 8.1 μIU/mL (normal range: 2.6–24.9 μIU/mL) and C-peptide level of 2.2 ng/mL (normal range: 1.1–4.4 ng/mL) prior to receiving dextrose. A 72-hour fast confirmed hyperinsulinemic hypoglycemia at 26 hours (glucose 36 mg/dL, insulin 10.3 mIU/L [normal range: 2.6–25 mIU/L], proinsulin 21.7 pmol/L [normal range: ≤21.7 pmol/L], C-peptide 2.3 μg/dL [normal range: 1.1–4.4 μg/dL], beta hydroxybutyric acid 0.06 mmol/L [normal range: 0.02–0.27 mmol/L], negative sulfonylurea screen, cortisol 28.9 μg/dL [normal range 6.2–19.4 μg/dL]). Serum calcium level was 8.8 mg/dL [normal range: 8.6–10.2 mg/dL] and parathyroid hormone level was 54 pg/mL [normal range: 10–65 pg/mL].

**Fig. 1:**
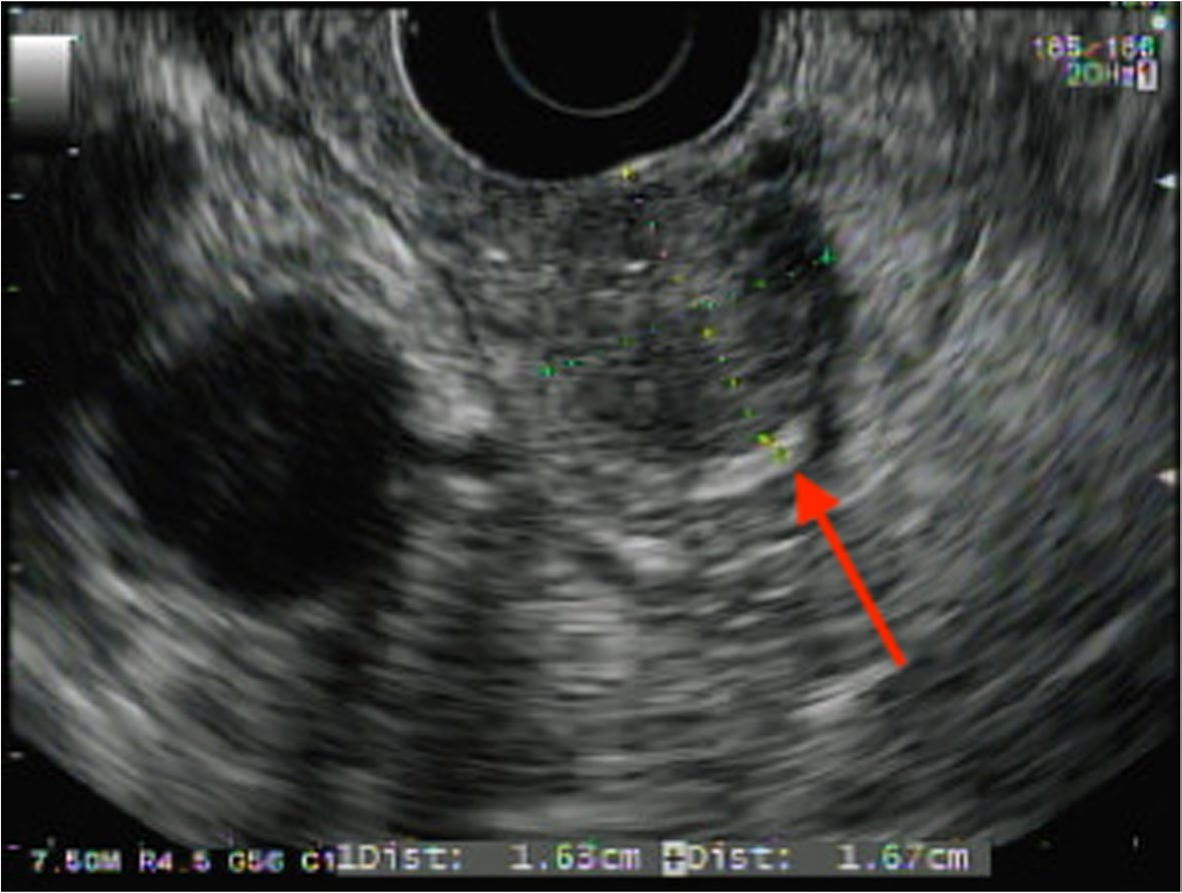
EUS 8/2013 – initial diagnosis. Arrow pointing to 1.6 cm × 1.6 cm duodenal mass

Abdominal CT with intravenous contrast showed a 1.1 cm × 1.6 cm × 2 cm hypervascular mass in the second portion of the duodenum without obstruction and a normal pancreas. OctreoScan did not show uptake in the area of the mass. Esophagogastroduodenoscopy identified a mass in the duodenal sweep and endoscopic ultrasound (EUS) showed a 1.6 cm × 1.6 cm duodenal mass (Fig. 1) with 3 hypoechoic well-defined lymph nodes distal to the mass measuring up to 1 cm in length. She underwent exploratory laparotomy with duodenotomy and NET enucleation in August 2013. Histopathology showed a well-differentiated intermediate grade 1.5 cm NET with Ki-67 index of 3–4%. All 4 of the 4 resected lymph nodes were negative for disease. Following surgery, she experienced weight loss of 30 pounds and denied any symptoms of hypoglycemia at her 6-month follow-up evaluation. The patient’s family history was negative for multiple endocrine neoplasia type 1 (MEN-1) syndrome, endocrine tumors or abnormalities of calcium metabolism, and her personal history was negative for nephrolithiasis or associated MEN-1 conditions. The patient’s calcium, parathyroid hormone, and prolactin levels were normal. MEN-1 testing was discussed with the patient but was not pursued at the time.

In September 2015 she reported hypoglycemia symptoms during exercise, and random glucose meter checks confirmed blood glucose levels ranging 60–70 mg/dL during these episodes. Seven-day continuous glucose monitoring (CGM) showed no glucose values < 60 mg/dL, and CT of the abdomen with intravenous contrast did not show evidence of disease. In 2015, Ga-68-DOTATATE imaging was not yet available at our institution to allow for further imaging evaluation.

**Fig. 2:**
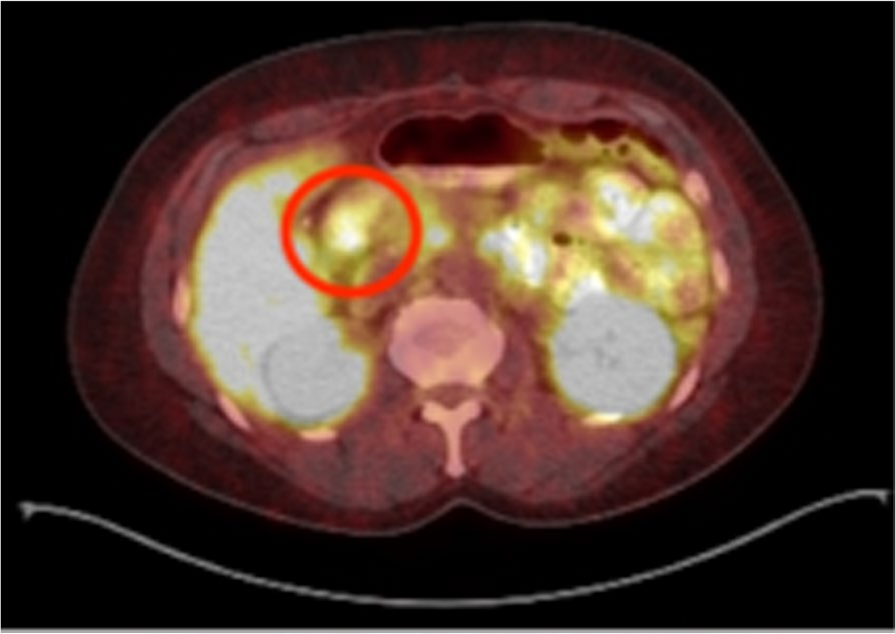
68-Gallium DOTATATE Scan 8/2017. Circle indicating 1.5 cm × 1.3 cm lymph node, signifying recurrent disease

In July 2017 the patient’s hypoglycemic symptoms returned with increasing frequency. Placement of another 7-day CGM showed that 29% of glucose values were < 60 mg/dL and revealed a pattern of nocturnal hypoglycemia. Abdominal CT evaluation did not show evidence of disease. PET-CT with Ga68-DOTATATE showed a 1.5 cm × 1.3 cm soft tissue nodule adjacent to the inferior pancreatic head and wall of the 2nd portion of the duodenum with maximum standardized uptake value (SUV) of 41 (Fig. 2). Repeated 72-hr fast was deemed unnecessary. EUS showed normal duodenum and pancreas but found peripancreatic lymphadenopathy. Lymph node fine-needle aspiration confirmed NET. Repeat surgery found metastatic, well-differentiated NET in 5 of 12 lymph nodes with a Ki-67 index of 3.8%. Insulin staining was not performed on the resected lymph nodes. Hypoglycemia resolved after surgery. Follow-up Ga68-DOTATATE scan in January 2018—4.5 years after her initial presentation—did not show evidence of disease. As of June 2021, she reported feeling well and CGM showed 99% time-in-range (blood glucose 70–180 mg/dL), with < 1% hypoglycemia over a 2-week period. Ga68-DOTATATE scan in 2020 showed small focus of uptake at the root of mesentery but was deemed possible overcall. Follow-up Ga68-DOTATATE scan in November 2021 showed no evidence of somatostatin receptor positive neoplasm, and diagnostic CGM is currently scheduled to occur every 3 months. Table 1 below is a summary of the patient’s history.

**Table 1:** Summary of Patient History

Year	Symptoms	Laboratory Workup	Diagnostic Imaging	Treatment/Surveillance
2013	Whipple’s triad, weight gain	• Serum glucose 49 mg/dL (normal range: 70–99 mg/dL), serum insulin 8.1 μIU/mL (normal range: 2.6–24.9 μIU/mL), C-peptide 2.2 ng/mL (normal range: 1.1–4.4 ng/mL) • 72-hour fast: hyperinsulinemic hypoglycemia	• Abdominal CT with intravenous contrast: 1.1 × 1.6 × 2 cm hypervascular mass in the second portion of the duodenum; normal pancreas. • OctreoScan: no uptake in the area of the mass. • EGD: mass in the duodenal sweep • EUS: 1.6 cm × 1.6 cm duodenal mass; 3 lymph nodes up to 1 cm in length.	Exploratory laparotomy with duodenotomy and NET enucleation
2015	Hypoglycemia during exercise	• Random glucose 60–70 mg/dL during episodes • 7-day CGM negative for hypoglycemia	Abdominal CT with IV contrast: no recurrence	
2017–2018	Increased frequency of recurrent hypoglycemia	7-day CGM with 29% nocturnal hypoglycemia	• Abdominal CT with IV contrast: no recurrence • PET-CT with Ga68-DOTATATE: 1.5 cm × 1.3 cm soft tissue nodule adjacent to inferior pancreatic head and wall of the 2nd portion of the duodenum, maximum SUV 41 • EUS: peripancreatic lymphadenopathy; normal duodenum and pancreas • Lymph node fine-needle aspiration: confirmed NET	• Exploratory laparotomy: 5/12 lymph nodes with well-differentiated NET • Hypoglycemia resolved after surgery
2020	Feeling well	14-day CGM: 99% time in range, < 1% time below range	Ga68-DOTATATE scan: small focus of uptake at the root of mesentery (possible overcall)	• Diagnostic CGM every 3 months
2021	Feeling well	14-day CGM: 98% time in range, < 1% time below range	Ga68-DOTATATE scan: no focal uptake	

## Discussion

Insulinoma is a rare tumor resulting in insulin hypersecretion. Patients with insulinoma present with neuroglycopenic symptoms such as confusion and seizure as well as occasional diaphoresis, anxiety, palpitations, and tremors. The majority of insulinomas are small (< 2 cm), single, sporadic, and arise within the pancreas with equal intra-organ distribution [1, 9, 10]. Ectopic insulinomas have also been described in the literature and have been reported to account for an estimated incidence of 1–2% of all insulinomas [11].

Ectopic insulinomas usually develop in ectopic pancreatic tissue and have been reported in 0.5–15% of autopsies as well as in 1 out of 500 abdominal surgeries [8]. In our patient, careful review of the initial resection specimen showed no evidence of adjacent ectopic pancreatic tissue.

A population-based study of 224 patients with surgically confirmed insulinomas presenting between 1927 and 1986 showed recurrence rates of 7% in patients with sporadic insulinomas compared with 21% in patients with MEN syndrome type 1 [12]. Recurrence of pancreatic insulinoma can develop 4–20 years after initial surgery [1], but there have not been reports of disease recurrence for extrapancreatic insulinomas. Also, previous cases of extrapancreatic insulinoma did not include significant follow-up evaluations as each respective patient was presumably cured surgically [2–8]. The longest follow-up evaluation reported among these cases was 4 months in the setting of a surgically-managed ectopic neuroendocrine tumor arising from the ligament of Treitz [8]. As our case seems to be the first recurrence of extrapancreatic insulinoma, the future surveillance and management of our patient remains a challenge Table 2.

**Table 2:** Summary of Extrapancreatic Insulinomas in the Literature

Year	Author(s)	Age (y)/ Sex	Symptoms and Duration	Location	Metastases	Localization Technique	Treatment	Follow up
2021	Zhang et al. [13]	23/male	1 year of recurrent hypoglycemia	Gastric antrum	Negative	• Pancreatic volume perfusion CT: positive • MRI abdomen: positive • Ga68-DOTATATE PET/CT: positive • 68 GA-Exendin-4PET/CT: positive • 18F-FDG PET/CT: positive • Endoscopic ultrasound: positive	Laparoscopic wedge resection of gastric antrum	N/A
2021	Wang et al. [14]	62/female	Postcoital bleeding initially; symptomatic hypoglycemia after 1 year	Cervical lip	Lungs and liver	• 18 FDG-PET: positive • Ultrasound: positive • CT: positive	Initially chemotherapy; palliative care with metastases	Patient died
2020	Garg et al. [15]	38/male	Recurrent hypoglycemia for 5 years, improved with food intake	Proximal jejunum	Negative	• Contrast-enhanced CT: positive • 68Ga-DOTATATE PET/CT: positive 68Ga-Exendin-4 PET/CT: positive	Excision	N/A
2016	Lombardi et al. [5]	79/male	Persistent hypoglycemia despite insulin cessation after 30 years with insulin-treated type 2 diabetes	Microscopically infiltrated appendix wall to sub serosa with lymphatic and vascular invasion	Lymphatic and vascular invasion; one lymph node	• Contrast-enhanced CT: negative • EUS: negative • 68Gallium DOTANOC: positive	Laparoscopic resection	2 months
2014	Ramkumar et al. [4]	44/female	Persistent hypoglycemia, diaphoresis, and palpitations, and seizures for 3 years, usually in the morning	Right kidney	Negative	• Abdominal CT: positive • MRI abdomen: positive • EUS: negative pancreatic lesion • 68Gallium DOTANOC: positive • 99mTc-HYNICTOC: positive	Abdominal exploration and right nephrectomy	3 months
2013	La Rosa et al. [2]	75/female	Vertigo, weakness, fainting, and loss of consciousness, improved with sugar intake	2nd portion of the duodenum	Negative	• Abdominal CT: positive • EGD: positive • EUS: positive	Surgically enucleated	32 months
2011	Xian-Ling et al. [3]	21/male	Persistent dizziness, sweating, and loss of consciousness for 3 years that resolved with glucose intake	Duodenohepatic ligament	Negative	• Transabdominal ultrasound: negative • Abdominal CT: positive • Digital subtraction angiography: positive	Laparoscopic resection	N/A
2009	Cárdenas et al. [6]	46/male	3 episodes of dizziness, blurred vision, headache, and confusion, improved sugar intake	Inferior splenic pole and hilum with perineural and vascular invasion	Three lymph nodes	• CT Scan: negative • EUS: negative • 185 MBq SC-99 m-Tc scintigraphy: negative • MRI abdomen: positive	Splenectomy	1 year
2005	Hennings et al. [8]	74/female	Intermittent dizziness for 1 year	Adjacent to ligament of Treitz	Negative	• MRI: negative • CT: negative • 111In-DTPA-octreotide: positive • EUS: positive • Transabdominal ultrasound: positive	Resection	4 months
1980	Yoshikawa and Wakasa [7]	76/female	16 years of upper abdominal pain, intense hunger, and loss of consciousness	Retroperitoneal adipose tissue	Negative	• Autopsy	Autopsy	Patient died

In patients with a biochemical diagnosis of insulinoma, surgical cure rates range from 77 to 100%. When technically possible, tumor enucleation is preferred. For tumors not amenable to enucleation, various surgical techniques can be pursued, such as segmental resection of the pancreas, distal pancreatectomy, or pancreaticoduodenectomy [16]. Complications associated with surgery include pancreatic fistula, pseudocyst, intra-abdominal abscess, pancreatitis, hemorrhage, and diabetes. For patients who are not surgical candidates or are awaiting surgical intervention, medical therapy and dietary modifications are important interventional measures. These include diazoxide or long-acting somatostatin analogs such as octreotide and lanreotide. Endoscopically directed ethanol ablation has been utilized in refractory individuals who are not surgical candidates, but this approach is not considered to be standard of care in healthier patients [17]. Typically, short-acting octreotide is initiated to assess for tolerability and side effects, and, if tolerated, can be transitioned to long-acting somatostatin analog therapy.

Malignant insulinomas are those that show evidence of local invasion into surrounding soft tissue or distant metastases to the liver or lymph nodes. The 10-year survival for malignant insulinomas is reported to be 29% [18]. Aggressive surgical resection including pancreatic resection is considered first-line surgical treatment in the setting of malignant insulinomas. Liver resection and even liver transplantation have been attempted to improve patient survival if hepatic metastases are present. When surgical interventions are not feasible, alternative debulking procedures such as radiofrequency thermoablation, cryotherapy, hepatic artery embolization and chemoembolization can be attempted; such options provide good but temporary palliation [19]. For patients with highly proliferating, rapidly progressing, or symptomatic insulinomas, chemotherapy may produce greater tumor size reduction compared with somatostatin analogues.

Everolimus, an oral mTOR inhibitor, has been used for malignant insulinomas associated with refractory hypoglycemia [20]. The exact mechanism by which everolimus can control hypoglycemia in patients with insulinoma is not fully elucidated, but mechanisms proposed include increasing peripheral insulin resistance as well as decreasing beta-cell proliferation, survival and metabolism [21]. Peptide receptor radiotherapy (PRRT), which uses radiolabeled somatostatin analogues to target specific peptide receptors on tumor cells is another treatment option being used in inoperable locoregional or distant metastatic gastreoenteropanceratic NETs [22]. 177Lu-labeled PRRT is currently the radionuclide of choice. PRRT for high-grade gastroenteropancreatic NETs has shown promising response rates, disease control rates, progression free survival, and overall survival [23]. In patients with metastatic insulinoma, PRRT is also effective in controlling hypoglycemia even in the setting of tumor regrowth [24]. New agents under investigation as treatment options for malignant insulinomas associated with refractory hypoglycemia include anti-insulin receptor monoclonal antibodies [25], oral somatostatin receptor drugs, and soluble stable glucagon [26].

As improvements in imaging studies continue to increase the frequency of pancreatic NET detection, recent studies have also evaluated the role of lymph node metastases in prognosis and management strategies. A retrospective review found tumor location, tumor size, Ki-67 index, and presence of lymphovascular invasion were associated with lymph node metastasis. Tumor diameter > 1.5 cm, tumors located in the head of pancreas, presence of lymphovascular invasion on surgical pathology, and Ki-67 index > 20% were reported to frequently present with lymph node metastasis [27]. Further analysis in this study showed that lymph node metastasis was significantly associated with a decrease in disease-free survival (4.5 years compared with 14.6 years for patients without lymph node metastasis). At this time, multiple studies report that both the number and presence of positive lymph nodes have important prognostic value in patients with pancreatic neuroendocrine tumors, thereby supporting the recommendation for systematic removal of lymph nodes in the peritumoral area during any pancreatic NET operation [28]. Patients with duodenal NETs treated with pancreaticoduodenectomy have been reported to have a higher incidence of metastasis to locoregional lymph nodes compared with patients with pancreatic NETs. In a study investigating surgical outcomes of patients with pancreatic versus duodenal NETs, those with duodenal NETs had more lymph node metastases. Yet, despite this difference in metastases, there was no impact on recurrence-free survival or overall survival between the two groups. Furthermore, those with duodenal NETs were more likely to have recurrent disease within 2 years of pancreaticoduodenectomy compared with those with pancreatic NETs. Further analysis is required to determine if these outcomes are similar in patients presenting with insulinomas. If so, this may suggest that a duodenal primary NET has higher malignant potential than a typical pancreatic primary NET [29].

At the present time, our patient continues to do well clinically with no evidence of significant hypoglycemia. She has declined octreotide treatment due to concerns with side effects and wishes to continue active surveillance with imaging. Given her recurrence of hypoglycemic symptoms and evidence of lymph node metastases 2 years after initial enucleation surgery, we suspect that she has microscopic, undetectable, clinically silent disease. As such, we will continue to monitor her closely for redevelopment of hypoglycemia.

## Conclusion

Extrapancreatic insulinomas are rare and the few cases of the tumors described in the literature do not report long-term follow-up assessment. This report presents the first known case of an extrapancreatic insulinoma recurrence and the longest reported followup. Current evidence shows that lymph node metastases of intrapancreatic insulinomas likely do not change prognosis. It is unclear if this is applicable to extrapancreatic insulinomas as well. More long-term outcome data are necessary to help determine how these patients should be monitored and managed. To monitor and manage extrapancreatic insulinoma recurrence we suggest patient education for hypoglycemic symptoms, monitoring for hypoglycemia with continuous glucose monitoring, annual imaging, and an ongoing discussion with patient regarding treatment with octreotide or alternative somatostatin receptor analog therapies.

## Data Availability

Data sharing is not applicable to this article as no datasets were generated or analyzed.

## Abbreviations

NET: Neuroendocrine tumor
EUS: Endoscopic ultrasound
MEN-1: Multiple endocrine neoplasia type 1
CGM: Continuous glucose monitoring
SUV: Standardized uptake value
PRRT: Peptide receptor radiotherapy
